# Performance of *Metarhizium rileyi* Nm017: nutritional supplementation to improve production and quality conidia

**DOI:** 10.1007/s13205-023-03911-6

**Published:** 2024-02-24

**Authors:** Cindy Mejía, Jaime Rocha, Johanna Sanabria, Martha Isabel Gómez-Álvarez, Ginna Quiroga-Cubides

**Affiliations:** 1 Centro de Investigación Tibaitatá, Corporación Colombiana de Investigación Agropecuaria-Agrosavia, Km 14 vía Mosquera-Bogotá, 250047 Mosquera, Colombia; 2 Departamento de Bioproductos, Corporación Colombiana de Investigación Agropecuaria-Agrosavia, Sede Central. Km 14 vía Mosquera-Bogotá, 250047 Mosquera, Colombia

**Keywords:** Fermentation, Conidia production, Quality control, Entomopathogen, Biocontrol

## Abstract

This study aimed to analyze the effect of nutritional supplements on improving conidia production of *Metarhizium rileyi* Nm017 at laboratory scale (yields of conidia/substrate and biomass/substrate, and substrate consumption). Also, the influence on quality parameters were evaluated (germination at 36 and 48 h, enzymatic activity, and insecticidal activity on *Helicoverpa zea*). Six treatments (T1–T6) were assessed and all of them reached maximum conidia concentration after 7 days fermentation, a feasible production timetable. Yields from treatment T6 (yeast extract + V8 juice) were 1.5–threefold higher than the other treatments. Conidia from T6 reached germinations of 56% and 12% at 36 and 48 h, respectively, higher than T1 (without supplements), which had the lowest values found. *M. rileyi* conidia obtained from treatment T6 had the highest enzymatic activity (0.45 U chitinase g^−1^, 0.28 U lipase g^−1^, and 1.29 U protease g^−1^). However, treatments with the highest conidia yields and enzymatic activity were not positively correlated to the efficacy against *H. zea.* When *M. rileyi* was produced on T5 (yeast hydrolysate + V8 juice), conidia were 35% more virulent than treatment T6. The findings evidenced the noticeable impact of nutritional substrate amended for conidia production and quality. This work showed the relevance of insecticidal activity assessment as a selection criterion in the mass production development of a biocontrol agent.

## Introduction

*Metarhizium rileyi* Farlow (previously known as *Nomuraea rileyi*) is a widespread dimorphic entomopathogenic fungus, used to control lepidopterans pests including more than 60 species such as *Anticarsia gemmatalis*, *Spodoptera frugiperda*, *Heliothis virescens*, and *Helicoverpa zea* (Boucias et al. 2016; Fronza et al. 2017; Liu et al. 2019). Its virulence is due in part to its high genetic variability, enzyme production, and stress tolerance that drive epizootics (Bertholdo et al. 2003; Boucias et al. 2016; Butt et al. 2016; Edelstein et al. 2005). *M. rileyi* is a very desirable microorganism as a biocontrol agent due to its narrow host specificity (Sinha et al. 2016).

The adoption of a microorganism as a biological control agent as part of integrated pest management will require more predictable performance and higher efficiency in propagule production (Lacey et al. 2001). Mass production of entomopathogenic fungi should show high versatility, require relatively low nutritional requirements for growth, and can be carried out using two main techniques: submerged fermentation and solid-state fermentation (Hölker and Lenz 2005; Mascarin et al. 2019). Submerged fermentation is preferred because of its high profitability, shorter production time, and easy control of process parameters. Nevertheless, propagules have a short life span and poor tolerance to adverse environmental conditions (Muñoz et al. 1995; Zaki et al. 2020; Jaronski 2023). Solid-state fermentation is the most used cultivation system for fungi, because it recreates the natural way they grow and produces aerial conidia (De la Cruz-Quiroz et al. 2016; Lara-Juache et al. 2021). These propagules are the main active ingredient in mycopesticides due to their process reproducibility, tolerance to abiotic stresses, infection performance, and easy mass production on a low-cost substrate (Hölker and Lenz 2005; Ibrahim et al. 2015; Mascarin and Jaronski 2016; Sala et al. 2019).

The major challenge for aerial conidia obtention is to stabilize its highly variable growth and low productivity by solid-state fermentation (Fronza et al. 2017; Iwanicki et al. 2020). The manipulation of culture medium composition positively alters conidia physiological growth to provide stability during fermentation and into formulation prototypes (Muñiz-Paredes et al. 2017; Zaki et al. 2020). Although the demonstrated virulence of *M. rileyi* against important economic insect pests, its high sensitivity to abiotic factors, poor sporulation, and demanding nutritional requirements have not allowed the development of an optimum culture medium for aerial conidia production (Boucias et al. 1984; Edelstein et al. 2005; Faria et al. 2017; Goettel and Roberts 1991; Grijalba et al. 2018). Among *Metahizium* species, concentrations of propagules have been reported in submerged fermentation (2 × 10^8^ blastospores mL^−1^ for *M. anisopliae*, 5 × 10^7^ blastospores mL^−1^ for *M. robertsii*, 7 × 10^7^ blastospores mL^−1^ for *M. rileyi*) and solid-state fermentation (1.2 × 10^9^ conidia g^−1^ for *M. anisopliae*, 3.9 × 10^9^ conidia g^−1^ for *M. rileyi*); demonstrating the highest productivity with solid systems (Grijalba et al. 2018; de Sá Santos et al. 2020; Iwanicki et al. 2021; Gotti et al. 2023). Furthermore, lethal times are shorter with aerial conidia than with blastospores for *Metarhizium* sp. (Gotti et al. 2023).

The selection of strategies to improve production efficiency is crucial for commercially developing biopesticides. Consequently, the screening of favorable and cost-effective conditions and additives to enhance conidial yield can expend considerable effort (Devi et al. 2001; Jaronski et al. 2023; Ravensberg 2011; Thakre et al. 2011). Moreover, quality specifications for biopesticides (microbiological, biochemical, and biological parameters) are necessary to ensure pre-determined quality and efficacy under the prescribed conditions for its use. Therefore, it is imperative to design a mass production to obtain fungal structures that withstand the downstream process and unfavorable field-application conditions, and provide consistent control of plant disease (Jeyarajan and Nakkeeran 2000). For solid-state fermentation, rice (high proportion of starch and amylase) is the most suitable substrate for quicker and better mass multiplication of *M. rileyi* (Thakre et al. 2011). Currently, *M. rileyi* conidia production systems are time-consuming (10 to 15 days) and conidial yields are unstable at times (2.2 × 10^6^ to 8.4 × 10^9^ conidia g^−1^ substrate) (Devi et al. 2001; Villamizar et al. 2004; Caro et al. 2005; Méndez et al. 2010; Thakre et al. 2011; Bich et al. 2018). Therefore, mass production of *M. rileyi* conidia is not sufficient to successfully incorporate it as a biological control agent in pest management.

Nutritional supplementation in a culture medium is the most effective way to improve quality conidia (germination, enzyme production, and virulence) in shorter fermentation times. For instance, the percentage of germination has been correlated with the addition of some substances, such as sugars and polyols (Hallsworth and Magan 1995; Jin et al. 1996), organic nitrogen sources (Caro et al. 2005; Devi et al. 2001), inorganic compounds (Aguirre et al. 2009; Jin et al. 1996), and cofactors (Elson et al. 1998; Jin et al. 1996). Likewise, substrate composition affects the production of virulence factors such as lipases, chitinases, and proteases. Therefore, the induction of these enzymes by different culture media could enhance biocontrol efficacy (Dhawan and Joshi 2017; Safavi et al. 2007; Mondal et al. 2016; Moon and Mun 2017).

Numerous studies on *Metarhizium* sp. production have demonstrated that specifically modifications of the nutritional environment impact significantly conidia development, pathogenicity, and conidial tolerance (Hallsworth and Magan 1995; Jackson and Jaronski 2009). However, to our knowledge, there is scarce information on how nutritional supplementation in *M. rileyi* cultures holistically affects its production, enzymatic activity, and virulence. Therefore, this study aimed to evaluate the effect of nutritional manipulation in the production of *M. rileyi* Nm017 on conidia quality (microbiological, enzymatic, and insecticidal activities) and process performance.

## Materials and methods

### Microorganism

The Colombian fungus strain used in this study was *Metarhizium rileyi*, encoded as Nm017, previously isolated from *Anticarsia gemmatalis* larvae and deposited at the Collection of Microorganisms with Interest in Biological Control of AGROSAVIA (Colombia), with an accession number 129. This isolate was cryopreserved at − 70 °C and propagated on MAYP (Edelstein et al. 2005) plus 0.1% w/v chloramphenicol (Colmed® International, Colombia), and incubated at 25 ± 0.5 °C for 7 days.

### Fermentation process

#### Preparation of culture media

**Fig. 1:**
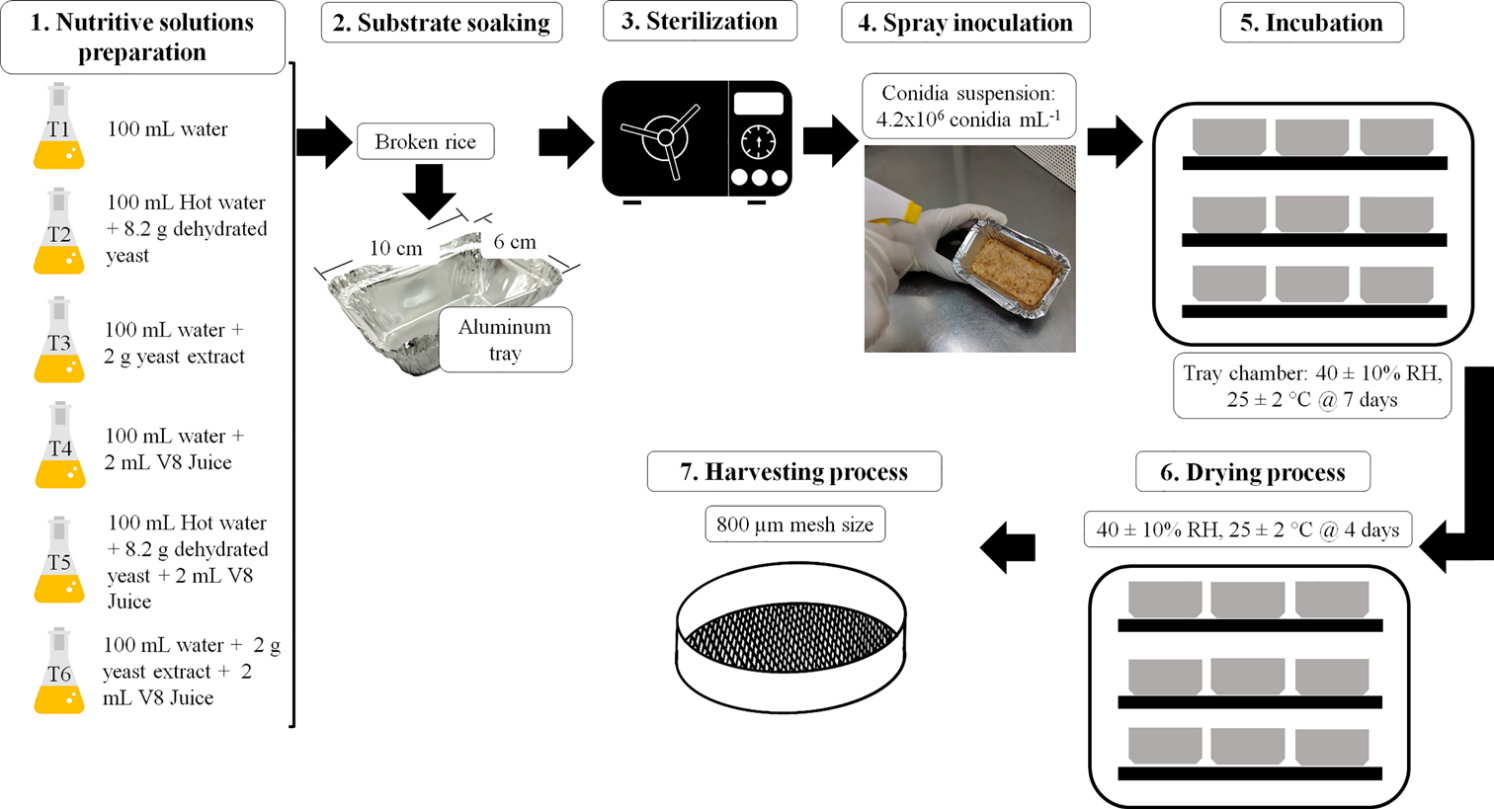
Schematic representation for *Metarhizium rileyi* Nm017 conidia production with substrate supplemented: 1. Nutritive solutions preparation: T1 (without supplements), T2 (8.2% v/v yeast hydrolysate), T3 (2% w/v yeast extract), T4 (2% w/v V8 juice), T5 (8.2% v/v yeast hydrolysate + 2% w/v V8 juice), and T6 (2% w/v yeast extract + 2% w/v V8 juice); 2. Substrate soaking: hydration with nutritive solutions; 3. Sterilization: 121 °C, 15 psi for 20 min; 4. Spray inoculation: conidia suspension adjusted to 4.6 × 10^6^ conidia mL^−1^; 5. Incubation: incubation room at 25 ± 2 °C and 70 ± 10% IHR, for seven days; 6. Drying process: incubation room at 25 ± 2 °C and 40 ± 10% EHR, for four days or until reaching a moisture content of ≤ 10%; 7. Harvesting process: vibratory sifting through a 800 μm mesh size

Five culture media supplements were evaluated for solid-state fermentation to produce aerial conidia at laboratory scale (Fig. 1). Each experimental unit had a mixture of broken rice supplemented with a nutritive solution (1:0.5 w/v), loaded into aluminum trays (10 cm × 6 cm × 3 cm), and subsequently sterilized at 121 °C, 15 psi for 20 min (TC-612, Gemmy Industrial Corp., China). The composition of the studied nutrient solutions was designed from three additives: yeast hydrolysate, yeast extract, and V8 juice. These were utilized in the following treatments (Fig. 1): T1, without supplements; T2, 8.2% v/v yeast hydrolysate, prepared with 100 mL hot water plus 8.2 g dehydrated yeast (Levapan®, Levapan S.A, Colombia; Caro et al. 2005); T3, 2% w/v yeast extract; T4, 2% w/v V8 juice; T5, 8.2% v/v yeast hydrolysate plus 2% w/v V8 juice; and T6, 2% w/v yeast extract plus 2% w/v V8 juice. Three replicated production sets were run for each experiment, with 15 experimental units per repetition.

#### Inoculation and incubation

The inoculum was prepared from *M. rileyi* Nm017 conidia grown for seven days on MAYP agar, as a conidia suspension in 0.1% v/v Tween® 80 solution (Millipore, Merck® KGaA, Germany). Conidia concentration was adjusted to 4.6 × 10^6^ conidia mL^−1^, and the suspension was sprayed in each substrate tray. Then, the trays were wrapped in a LLDPE film (porosity 45.2%) and placed in an incubation room for 7 days, at a temperature of 25 ± 2 °C and internal relative humidity (IRH) of 70 ± 10% (Fig. 1).

### Drying process and conidia recovery

After inoculation, the weight of the substrate for each experiment was measured. After fermentation, each tray was covered with a porous cellulose membrane, placed in a room at 25 ± 2 °C and external relative humidity (ERH) of 40 ± 10%, for 4 days to enhance hyphal maturation, sporulation, and drying until reaching a moisture content of ≤ 10%. *M. rileyi* Nm017 (Fig. 1). Spore powder from the dried substrate was harvested by vibratory sifting through a mesh sieve (800 μm mesh size; U.S. Standard sieve series No. 50, The W. S. Tyley Company, U.S.A.). The weight and moisture content of the conidial powder collected were measured.

### Conidial germination

Germination was assessed from each culture medium, using the recovered dry conidia (conidial powder). The microbiological parameter percentage of germination at 36 and 48 h was assessed. For each treatment, 1 g samples picked up aseptically were diluted in 0.1% v/v Tween® 80 solution, and decimal dilutions were done until 10^–2^. An aliquot of 100 µL of 10^–2^ dilution was plated on water agar plus 2% w/v yeast extract (Difco®, Thermo Fisher Scientific, U.S.A.), 2% v/v V8 juice (V8® Vegetable Juice, Campell’s, U.S.A.), and 0.00008% w/v benomyl [Benlate 50% w/w (WP), DuPont, Spain]. The Petri plates were incubated at 25 ± 2 °C for 36 and 48 h, and the germ tube growth was stopped with a lactophenol blue solution at each time (Sigma-Aldrich®, Merck KgaA, Germany). Germinated and non-germinated conidia were read in an optical microscope (400X magnification; CH30, Olympus®, Japan), and at least 100 conidia were scored per replicate. Conidia was considered germinated when its germ tube was at least twice its diameter (Ekesi et al. 1999; Milner et al. 1991). The results were reported as a percentage of germination at 36 and 48 h.

### Performance parameters

The conidia/substrate yield was evaluated by counting microscopically (400X magnification; CH30, Olympus®, Japan) with a Neubauer hemocytometer (BOE 01, Boeco, Germany) from the dilutions used in conidial germination. The moisture content of the substrate samples was also determined using a halogen balance (MLS 50–3, Kern, Germany) at 121 °C to determine the moisture content (% kg water kg^−1^ dry material). Subsequently, concentrations were corrected as conidia per dry substrate gram (conidia g^−1^).

Fungus colonized substrate, conidial powder, and substrate after harvesting from each tray and treatment was taken to estimate the biomass/substrate yield (g biomass per kg dry substrate, g kg^−1^) (Shay et al. 1987). The percentage of the consumed substrate in each tray was calculated as the difference between weight of the dry substrate before inoculation and after harvesting (Tumuhaise et al. 2018).

### Enzymatic activity

Conidia crude extracts were prepared as described by Villamizar et al. (2001), with modifications. 1 g of the colonized dried substrate of each treatment was suspended in 10 mL 1% v/v Tween® 80 solution and stirred at 3000 rpm for 1 h at room temperature. Suspensions were centrifuged for 10 min at 4000 rpm to obtain the supernatant, which was used in enzymatic assays.

Lipase activity was determined according to Beys da Silva et al. (2010) and Glogauer et al. (2011). 20 µL of crude extract were mixed with 230 µL of substrate [3 mg *p*-nitrophenyl palmitate, *p*NPP (Sigma®, Merck KgaA, Germany)] in 1 mL isopropanol (Sigma®, Merck KgaA, Germany) and 9 mL 50 mM Tris–HCl pH, containing 40 mg Triton X-100 (Sigma®, Merck KgaA, Germany) and 10 mg Arabic gum (Sigma®, Merck KgaA, Germany). The resulting solution was incubated at 37 ± 1 °C for 30 min. Absorbance was measured at 400 nm, and released *p*-nitrophenol was estimated using a standard curve. One unit of the enzyme was defined as the amount of enzyme that released 1 µmol *p*-nitrophenol per minute.

Chitinase activity was measured using 20 µL crude extract and 100 µL p-nitrophenyl-N-acetylglucosamine (Sigma®, Merck KgaA, Germany) (1 mg mL^−1^ in citrate buffer 0.1 M pH 5). The mixture was incubated at 37 ± 1 °C for 30 min and stopped with 150 µL NaOH-glycine pH 10.4 (Sigma®, Merck KgaA, Germany). Absorbance was measured at 400 nm, and the amount of *p*-nitrophenol was estimated using a standard curve. One unit of the enzyme was defined as the amount of enzyme that released 1 µmol p-nitrophenol per minute (Mejía et al. 2020).

Protease activity was determined using casein (Sigma®, Merck KgaA, Germany) at 0.65% as substrate. The reaction mixture contained 130 µL of 0.65% w/v casein and 25 µL of crude extract. The reaction was incubated for 10 min at 37 ± 2 °C, and stopped by adding 130 µL of 110 mM trichloroacetic acid (Sigma®, Merck KgaA, Germany), with incubation at 37 ± 2 °C for 20 min. The mixture was centrifuged at 10,000 rpm, for 15 min. 250 µL supernatant was mixed with 625 µL 500 mM of sodium carbonate (Sigma®, Merck KgaA, Germany) and 125 µL of 0.5 M Folin–Ciocalteu (Sigma®, Merck KgaA, Germany) solution. The mixture was incubated 30 min at 37 ± 2 °C. The absorbance was measured at 660 nm. One unit of the enzyme was defined as the amount of enzyme to release 1 µmol tyrosine per minute (Cupp-Enyard 2008).

### Insecticidal activity

#### Insect rearing

The larvae of *Helicoverpa zea* were reared on an artificial diet prepared according to Greene et al. (1976) with modifications (Gómez et al. 2010), by Rearing Unit of AGROSAVIA – Tibaitatá Research Center. The rearing was in a controlled environment room at 28 ± 1 °C and a photoperiod of 12 h light/darkness.

#### Bioassay

Bioassays were carried out with second-instar larvae of *H. zea*, following the methodology described by Mejía et al. (2020). Larvae dorsum was inoculated by applying 2 µL of fungal suspension prepared in 0.1% v/v Tween® 80 solution and concentration adjusted to 1 × 10^6^ conidia mL^−1^. Control larvae were inoculated with 0.1% v/v Tween® 80 solution. Inoculated larvae (sampling unit) were individually transferred to 15 mL plastic cups containing one maize grain as a feeding substrate. Twelve cups were placed in a 473 mL plastic box (experimental unit) and incubated under controlled conditions (25 ± 2 °C, 60% ERH) and a light/darkness photoperiod of 12 h. Larval mortality was recorded daily for 10 days. Each treatment had three replicates (3 experimental units) for a total of 36 larvae per treatment. The percentage of corrected mortality (treatment mortality corrected by mortality in the control treatment) was calculated using the Schneider-Orelli formula (Zar 1999).

### Data analysis

The experiments in this study had three repetitions in time and three replicates per treatment. For statistical purposes, germination and efficacy were arcsine or square root transformed, and conidia concentration was log10 transformed. The results were verified for data normality (Shapiro–Wilk test) and the homoscedasticity (Bartlett's test). Statistical significance of the results was determined using one-way analysis of variance (ANOVA), and mean comparison test (Tukey HSD; confidence level of 95%), using Minitab® 19 Statistical Software (Minitab®, LLC, USA).

## Results and discussion

### Fermentation and drying processes

**Fig. 2:**
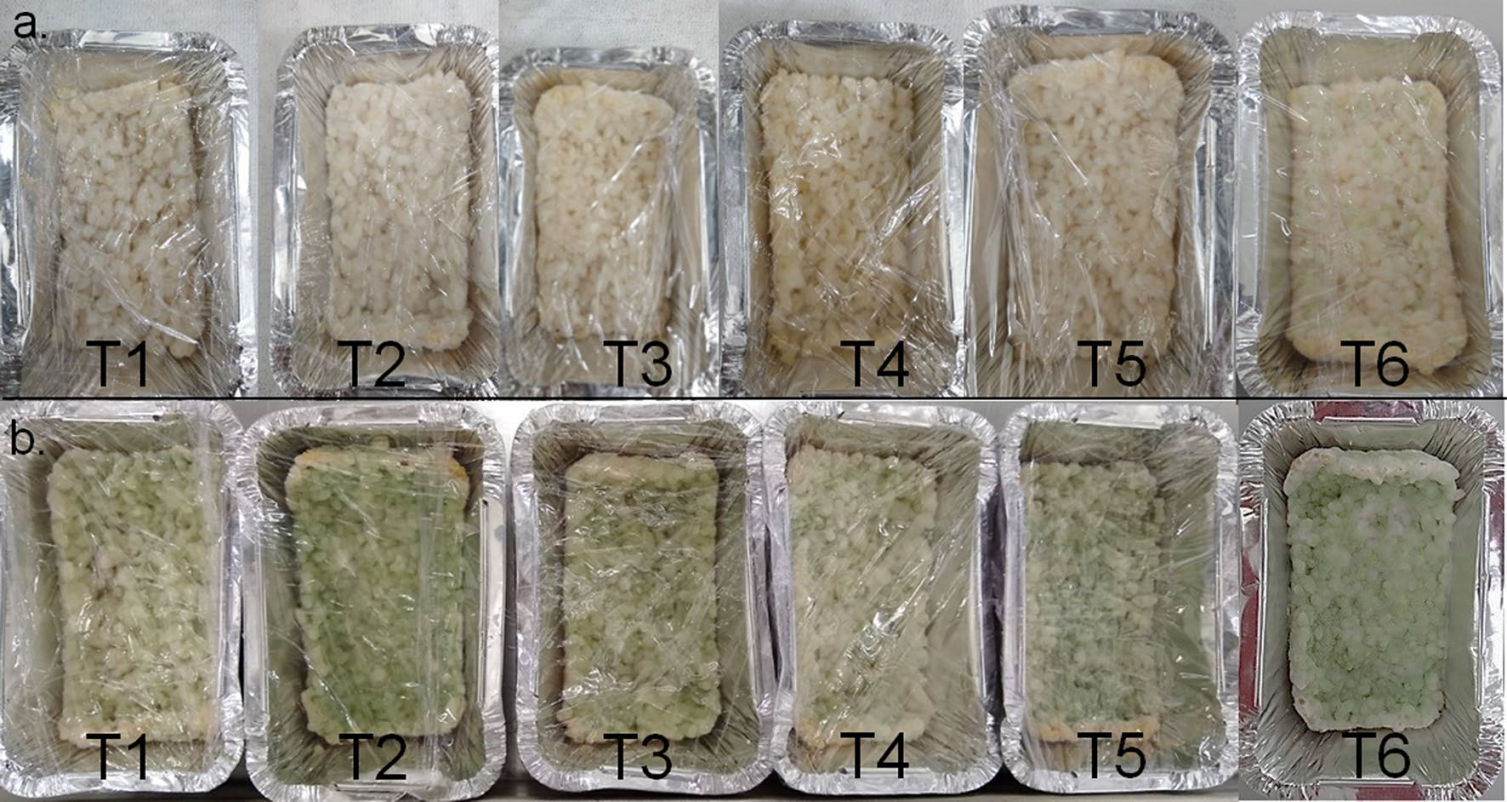
Process fermentation of *Metarhizium rileyi* Nm017 with substrate supplemented (T1, without supplements; T2, 8.2% v/v yeast hydrolysate; T3, 2% w/v yeast extract; T4, 2% w/v V8 juice; T5, 8.2% v/v yeast hydrolysate + 2% w/v V8 juice; and T6, 2% w/v yeast extract + 2% w/v V8 juice): **a** Third day of fermentation; **b** Seventh day of fermentation

*Metarhizium rileyi* Nm017 grew and sporulated in all of the treatments assessed. The fermentation started with the colonization of white mycelium during the first 3–4 days for treatments T1 through T5 (Fig. 2a), and then production of olive-green aerial conidia started within 5–7 days for all media (Fig. 2b). However, T6 (2% w/v yeast extract + 2% w/v V8 juice) culture medium showed mycelium as early as the second day, and patches of conidia on its mycelial mat on the fourth day (Fig. 2). Different published protocols of mass multiplication have used yeast extract to increase aerial conidia production (Jaronski 2023; Mishra et al. 2016). Also, Devi et al. (2001) reported that yeast extract is required for the fungi mycelial growth. On the other hand, clarified V8 Juice at agar produces substantially more conidia in less time for *Fusarium* sp. strains (Elson et al. 1998). Moreover, these two nutritional supplements provided cheap carbon and nitrogen sources to boost better fungal growth (Jaronski 2023). Therefore, for a fungus such as *M. rileyi* with slow growth and sporadic sporulation on solid media, the nutrition augmented with yeast extract and V8 juice significantly influenced conidiation, and their action allowed increasing the effect that each one had individually (treatments T3 and T4).

All nutritional supplementation proposed herein shorted the culture time of Nm017 to 7 days, traditionally ranging from 10 to 15 days (Devi et al. 2001; Villamizar et al. 2004; Caro et al. 2005; Méndez et al. 2010; Thakre et al. 2011; Bich et al. 2018). During the drying process, the loss of surface water over time caused the volume reduction of the fermentation solid matrix and, probably, the aerial conidia, which made the conidia dustier and less linked to the substrate.

### Conidial germination

**Fig. 3:**
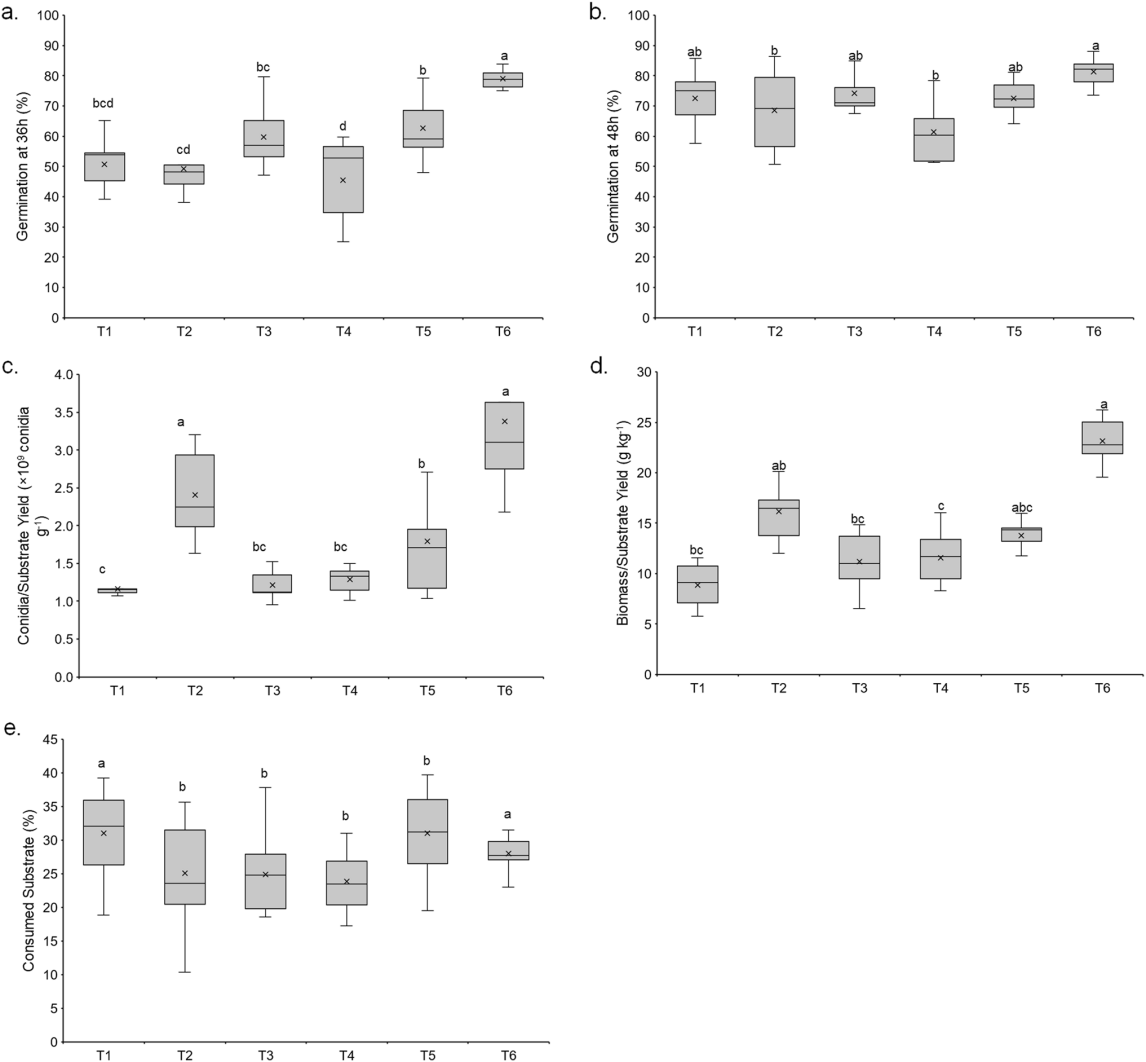
Conidial germination and performance parameters of *Metarhizium rileyi* Nm017 conidia obtained from substrate supplemented (T1, without supplements; T2, 8.2% v/v yeast hydrolysate; T3, 2% w/v yeast extract; T4, 2% w/v V8 juice; T5, 8.2% v/v yeast hydrolysate + 2% w/v V8 juice; and T6, 2% w/v yeast extract + 2% w/v V8 juice): **a** Germination at 36 h (% conidia germinated per total conidia); **b** Germination at 48 h (% conidia germinated per total conidia); **c** Conidia/substrate yield (conidia g^−1^ dry substrate); **d** Biomass/substrate yield (g biomass kg^−1^ dry substrate); **e** Consumed substrate (% kg substrate consumed per kg^−1^ initial substrate). Treatments with no common letters are significantly different according to Tukey HDS test (*α* = 95%). Means are represented with the symbol ×

Germinations of conidial powder from each culture medium assessed at 36 h and 48 h were less than 80%, except conidia from T6 (Fig. 3a and b). The findings showed that the three nutritional sources and their interaction were significantly relevant for germination at 36 h; while germination at 48 h was affected significantly by yeast extract and V8 juice (Germination at 36 h: *F*_*5,48*_ = *16.6, p* < *0.0001*; Germination at 48 h: *F*_*5,48*_ = *4.84, p* = *0.0012*). Early germination at 36 h was defined by Faria et al. (2017) as conidial vigor, which relates to the strength of conidia germination and germ tube growth. Vigor is strongly influenced by the fermentation system and downstream processing. For instance, rice substrate has been supplemented with additives to increase sporulation and germination, unlike substrates such as barley, oats, or wheat, probably due to their nitrogen and micronutrient content (Jaronski 2023). Likewise, rice contains a higher content of starch and amylase, and its hydrolysis produces glucose and maltose. Maltose released by fungal enzymes induces a series of mechanisms to accelerate fungi multiplication, including germination for mycelium production and subsequent sporulation (Thakre et al. 2011). Therefore, the substrate and additives used must provide a high availability of nutrients over a large surface area to promote germination and conidia formation (Machado et al. 2010; Mascarin et al. 2010).

The germination at 36 h revealed that Nm017 conidia production requires slower drying to maintain high conidial viability, as was demonstrated with other *Metarhizium* sp. (Jaronski and Jackson 2012). Moreover, a previous evaluation to this study using Nm017 strain and T1 medium (without supplements), demonstrated the protective effect of the slower drying process. It was found that the colonized substrate had an initial moisture content of 54.4% and an initial conidial germination at 48 h of 81.1%, and after 4 days of drying, the substrate ended up with a moisture content of 8.9% and a conidial germination of 78.6% (unpublished data). Hence, the reduction in germination was only 3%.

The greatest increase in germination among the two evaluation times was observed with treatment T1 (30%), while the smallest increase was observed with T6 (3%). Thus, the highest and most vigorous conidia were those obtained in the T6 substrate. The V8 juice micronutrient source used in T4, T5, and T6 is rich in trace elements, vitamins, and amino acids, which are essential cofactors that stimulate the metabolic processes involved in germination (Elson et al. 1998; Jin et al. 1996). It also has a remarkable calcium content (16 mg 100 mL^−1^), which has been shown to modulate the growth rate of the germ tube, *i.e.*, it is a precursor to the hyphae extension and medium colonization (Aguirre et al. 2009; Jin et al. 1996). Furthermore, Ca^2+^ is a signalling molecule involved in tolerance to stress conditions and virulence. Six major types of Ca^2+^ transporters have been reported for homeostasis and signalling (Roy et al. 2020).

### Performance parameters

The highest values of the process parameters assessed were reached with the T6 treatment (Fig. 3c, d, and e). All treatments had a notable impact on conidia/substrate yield, in which T6 and T2 showed the highest and most significant values (Fig. 3c; *F*_*5,48*_ = *21.1, p* < *0.0001*), while biomass/substrate yields were statistically different between treatments (Fig. 3d; *F*_*5,48*_ = *41.3, p* < *0.0001*). However, the variable substrate consumption demonstrated equitable nutritional use of culture medium (24–31%), without significant differences between media (Fig. 3e; *F*_*5,48*_ = *2.22, p* = *0.0678*).

The culture medium without extra sources, T1, did not show significant sporulation (Fig. 3c), which may be due to the lack of suitable extra nutritional sources for conidiogenesis (Caro et al. 2005; Keppanan et al. 2018; Kumar et al. 2011; Webb and Manan 2017). On the contrary, the massive growth of *M. rileyi* Nm017 observed in treatments T2 (8.2% yeast hydrolysate) and T6 (2% yeast extract plus 2% V8 juice) stand out, possibly due to the presence of organic nitrogen sources, recognized as essential for mycelial growth and conidiogenesis (Devi et al. 2001). Although T2 contained a germination promoter (Caro et al. 2005) and showed significant effects over yields, its sporulation and germination had significant differences compared to T6 (Fig. 3a–c).

The commercial viability of potential biopesticides is defined by their suitability for mass production. Values achieved with both media, T2 and T6, were higher than those observed with other commercial entomopathogenic fungus, *e*.*g.*, *M. rileyi* strains growth on a medium rich in soybeans (1.5 × 10^9^ conidia g^−1^ substrate; Caro et al. 2005), wheat bran enriched with malt soda (3.0 × 10^9^ conidia g^−1^ substrate; Villamizar et al. 2004), and crushed sorghum (1.4 × 10^9^ conidia g^−1^ substrate; Devi et al. 2001). Also, Nm017 production presented appropriate biomass yields for commercial entomopathogenic species, like *M. anisopliae* (27–42.2 g kg^−1^) and *B. bassiana* (20–40 g kg^−1^) (Keppanan et al. 2018; Tumuhaise et al. 2018; Liu et al. 2015; Pham et al. 2010; Posada-Flórez 2008; Renuka et al. 2015). This could be due to the substrate used in this study (rice), in which most fungi reach the highest biomass production (Gouli et al. 2013; Li et al. 2010; Jaihan et al. 2016; Saldarriaga et al. 2017). Moreover, *M. anisopliae* isolates and Nm017 had similar substrate consumption patterns, *e.g.*, Tumuhaise et al. (2018) reported a consumption percentage of 32.8%, and Agbessenou et al. (2021) reached values close to 25%.

### Enzymatic activity

**Fig. 4:**
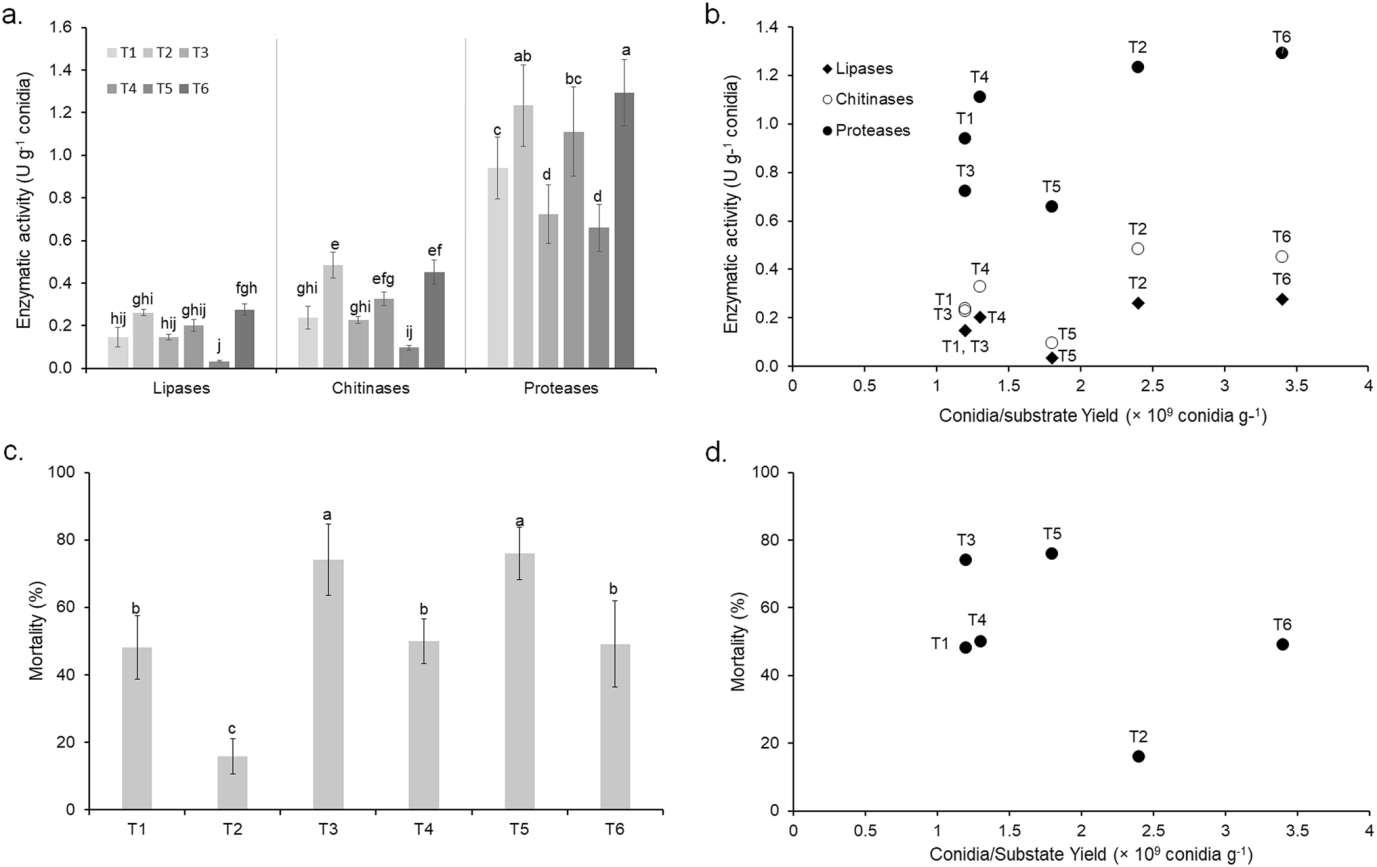
**a** Enzymatic activity of *Metarhizium rileyi* Nm017 conidia obtained from substrate supplemented (T1, without supplements; T2, 8.2% v/v yeast hydrolysate; T3, 2% w/v yeast extract; T4, 2% w/v V8 juice; T5, 8.2% v/v yeast hydrolysate + 2% w/v V8 juice; and T6, 2% w/v yeast extract + 2% w/v V8 juice); **b** Conidia/substrate Yield (conidia g^−1^ dry substrate) vs. Enzymatic activity (U g^−1^ conidia); **c** Mortality against *Helicoverpa zea* second instar larvae caused by *Metarhizium rileyi* Nm017 conidia obtained from substrate supplemented (%); **d** Conidia/substrate Yield (conidia g.^−1^ dry substrate) vs. Mortality (% dead larvae per total larvae). Mean values (± SD) followed with no common letters are significantly different according to Tukey HDS test (*α* = 95%)

Similarly, fungal enzymes produced on solid-state fermentation have been highly regulated by the availability of carbon and nitrogen, pH, relative humidity, moisture content, and temperature, among other factors (Mondal et al. 2016; St. Leger and Wang, 1998). In this research, yeast hydrolysate, yeast extract, and V8 juice with vitamin content increased the three groups of evaluated enzymes (lipase, chitinase, and protease activity), and improved the conidial yield. Among the enzymatic activities evaluated, *M. rileyi* produced a higher level of proteases compared to chitinases and proteases (*F*_*17,144*_ = *249, p* < *0.0001).* The lipase activity was between 0.03 and 0.15 U g^−1^ in all of the treatments except T5, which was the lowest value found. The chitinase activity was substrate dependent in mass production (Fig. 4a) and the highest value was obtained in conidia produced on substrates T2 and T5 (0.10 and 0.23 U g^−1^, respectively). Similar to lipases, the lowest chitinase activity was measured in treatment T5. Significant differences were found in the protease activity of conidia produced on the six substrates in solid-state fermentation, with values between 0.77 and 1.3 U g^−1^. The highest protease activity was reached with treatments T2 and T6, like chitinases, and the lowest values were found with treatments T3 and T5.

The results analysis showed that the enzymatic activity was affected by the nitrogen and micronutrient supplementation (Fig. 4b). Lipase activity was influenced by the three nutritional sources (yeast hydrolysate, yeast extract, and V8 juice) and their interactions, whereas the protease activity was influenced by yeast extract, and V8 juice. The chitinase activity only presented relevant effects with nitrogen and micronutrient interaction. Overall, T2, T4, and T6 supplementation affected both conidia yield and enzymatic activity (Fig. 4b). Comparably, Ferreira et al. (2021) showed that culture medium supplemented with sodium nitrate and riboflavin enhanced protease activity and conidia production of *Metarhizium robertsii*. Similarly, additives such as soybean protein combined with wheat bran induced the protease activity of *M. anisopliae* (Kim et al. 2020), and the supplementation with yeast extract improved the chitinase composition of *M. anisopliae* (Dhar and Kaur 2009). Also, it was demonstrated that C:N ratio is a crucial variable that induces enzyme expression, *e.g.*, the use of potato flour substrate with a C:N ratio of 30:1 increased the enzymatic activity of *B. bassiana*, compared with rice powder substrate with a C:N of 10:1 (Mejía et al. 2020). Likewise, an isolate of *B. bassiana* produced greater protease Pr1 activity in solid media with a C:N ratio of 10:1 (Safavi et al. 2007).

### Insecticidal activity

The use of supplements as enhancers of virulence has been tested before for entomopathogenic fungi. For instance, *B. bassiana* conidia harvested from wheat bran, rice bran, and SDAY reached the highest mortalities on the browntail moth *Euproctis chrysorrhoea*, compared to conidia obtained from other nutritional substrates such as millet, rice paddy, wheat, rice, and corn flour (Bena-Molaei et al. 2011). The nutritional composition of substrates could induce several virulence factors related to larvae mortality, *e.g.*, proteins such as hydrophobins and adhesins that mediate conidia attachment to cuticle insect surface. (Butt et al. 2016; Sevim et al. 2012; Schrank and Vainstein 2010).

Although enzymes have been considered crucial virulence factors of entomopathogenic fungi, and several authors described the relatedness between enzymes and mortality (Dhawan and Joshi 2017; Gebremariam et al. 2022; Pelizza et al. 2012; Svedese et al. 2013), we did not find correlation among the high enzymatic activity of *M. rileyi* and the uppermost insecticidal activity against *H. zea*. In this study, conidia with the lowest levels of enzymes caused higher mortalities of *H. zea*. Accordingly, treatments T3 (2% yeast extract) and T5 (8.2% yeast hydrolysate + 2% V8 juice) with low levels of enzyme activity, significantly influenced the insecticidal activity of *M. rileyi* against *H. zea* second instar larva (Fig. 4c; *F*_*5,53*_ = *50.2, p* < *0.0001*). The lowest efficacy (18%) was found in conidia from T2. Efficacies between 48 and 50% were obtained with conidia produced in treatments T1, T4, and T6, with no significant differences between them. The highest values of efficacy were related to yeast extract and sources interaction effects (Fig. 4c). Also, Petlamul and Prasertsan (2012) characterized several *Beauveria* and *Metarhizium* isolates based on their germination rate, conidia production, radial growth, enzyme activity, and virulence against *Spodoptera litura*. They found that a strain of *B. bassiana* had the highest germination rate and was the most virulent, but the lowest enzymatic activity. On the other hand, *M. anisopliae* strains produced the highest chitinase and protease activities.

These results suggest that the nutritional composition could be inducing other relevant virulence factors during fungal attachment and colonization of the insect. For instance, nutrients and fermentation system have been reported to influence the expression of different virulence factors such as collagen-like protein to evade the insects´ immune response, trehalases to use trehalose from the hemolymph, and regulation of heat-shock proteins and enzymes involved in response to oxidative stress (Gotti et al. 2023; Iwanicki et al. 2023). Pang et al. (2023) evidenced that the virulence of *M. rileyi* against *S. frugiperda* was determined by both expression of protective and detoxifying enzymes from the host and resistance to oxidative stress of the fungus.

## Conclusions

Through the research, we have demonstrated the effects of nutritional supplementation of the substrate on production and quality of *M. rileyi* Nm017 conidia. Results showed that the designed nutritional enrichment strategies enhanced the fungal viability and enzymatic activity. However, no nutritional relationship was found between conidia quality, enzyme activity, and biological activity. These findings supported the relevance of including the insecticidal activity for production strategies of potential biocontrol microorganisms as a selection criterion, in addition to germination and productivity. Further research is required to determine different virulence factors associated with insecticidal activity and production development on higher scales.

## Author contribution statement

CM: conceptualization, investigation, methodology, formal analysis, writing—original draft, writing—review and editing; JR: investigation, methodology, formal analysis, writing—review and editing; JS: investigation, methodology, formal analysis, writing—original draft; MIG-Á: methodology, writing—review and editing, supervision, project administration, funding acquisition; GQ-C: conceptualization, investigation, methodology, formal analysis, writing—original draft, writing—review and editing, supervision.

## Data Availability

The data sets generated and analyzed in this study are available from the corresponding author on reasonable request.
